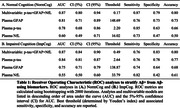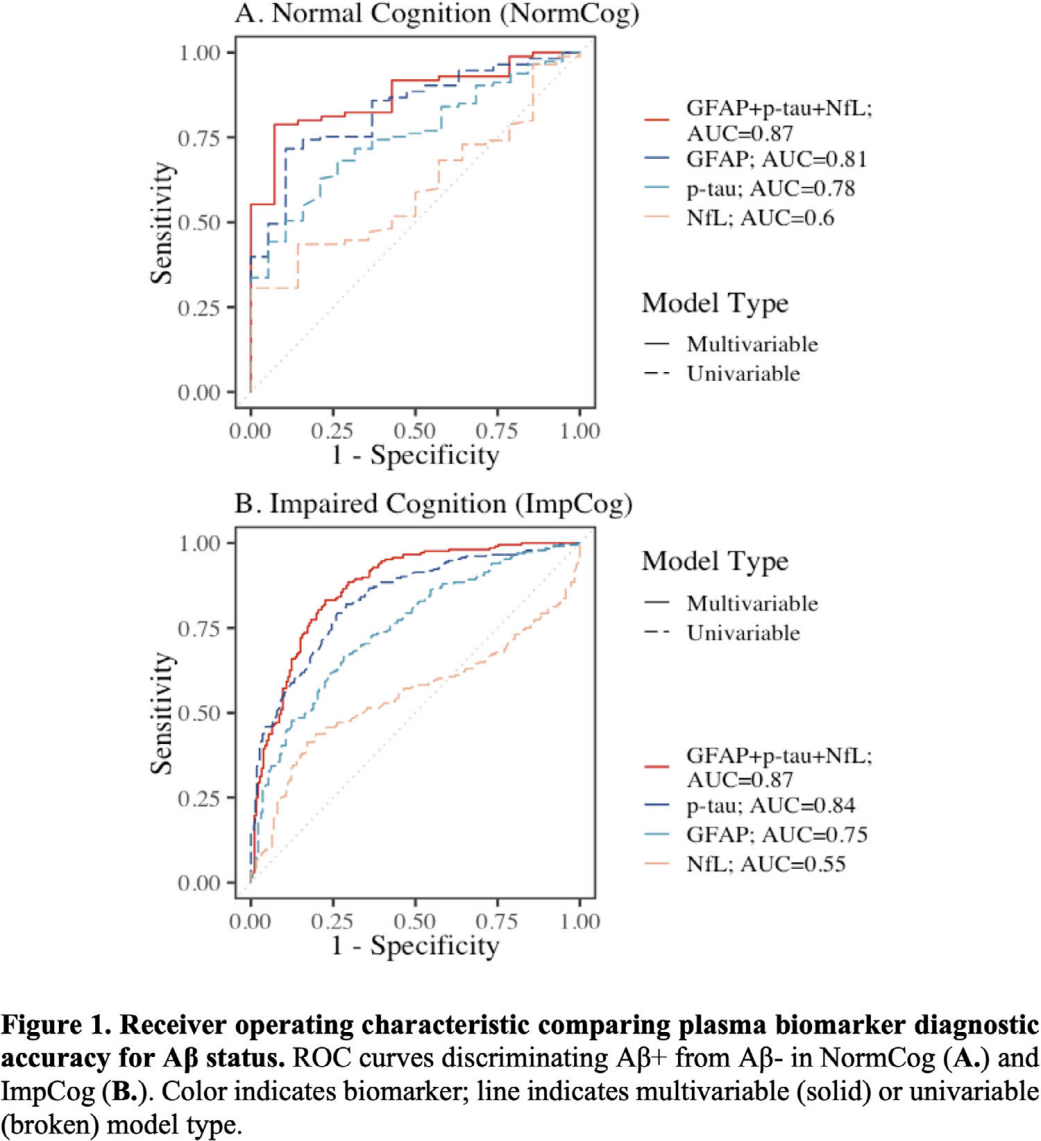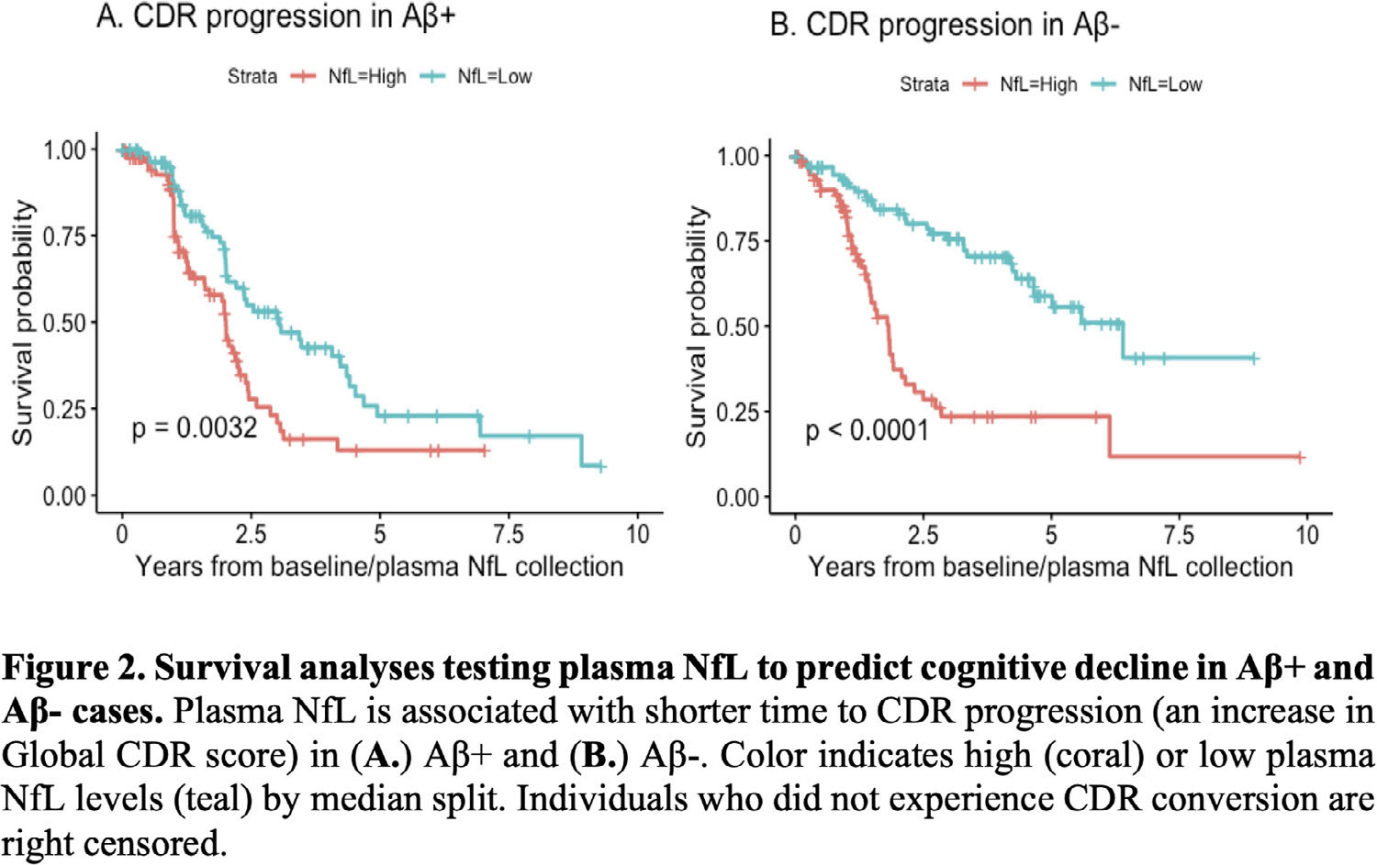# Combining plasma biomarkers for β‐amyloid diagnosis and cognitive prognosis across the clinical spectrum: normal, amnestic, and non‐amnestic

**DOI:** 10.1002/alz.085075

**Published:** 2025-01-09

**Authors:** Katheryn A Q Cousins, Jeffrey S Phillips, Sandhitsu R. Das, Kyra O’Brien, Thomas F. Tropea, Alice Chen‐Plotkin, Leslie M. Shaw, Ilya M. Nasrallah, Dawn Mechanic‐Hamilton, Corey T McMillan, David J Irwin, Eddie B Lee, David A Wolk

**Affiliations:** ^1^ Department of Neurology, University of Pennsylvania, Philadelphia, PA USA; ^2^ Perelman School of Medicine, University of Pennsylvania, Philadelphia, PA USA; ^3^ University of Pennsylvania, Philadelphia, PA USA; ^4^ Dept of Pathology & Laboratory Medicine, University of Pennsylvania, Perelman School of Medicine, Philadelphia, PA USA; ^5^ Centre for Biomedical Image Computing and Analytics, University of Pennsylvania, Philadelphia, PA USA; ^6^ Penn Alzheimer’s Disease Research Center, University of Pennsylvania, Philadelphia, PA USA

## Abstract

**Background:**

Alzheimer’s disease (AD) is clinically heterogeneous, and can manifest as amnestic or non‐amnestic dementia, or be pre‐manifest in persons with normal cognition (NormCog). Utility of plasma biomarkers to diagnose AD and prognose cognitive decline must be evaluated in a clinically heterogeneous population representative of the AD spectrum. We investigate pathological and cognitive correlates of plasma phosphorylated tau 181 (p‐tau_181_), glial fibrillary acidic protein (GFAP), and neurofilament light chain (NfL) across AD and AD‐related dementias (ADRD). We test 1.) if combing biomarkers improves diagnostic accuracy for β‐amyloid (Aβ) in NormCog and impaired cognition (ImpCog), 2.) if Aβ diagnostic accuracy in ImpCog differs by clinical syndrome (amnestic dementia vs. non‐amnestic dementia vs. mild cognitive impairment [MCI]), and 3.) how biomarkers predict future cognitive decline in Aβ+ and Aβ‐.

**Methods:**

Participants were NormCog (n=132) and ImpCog (n=461; 130 MCI/impairment without MCI; 61 amnestic; 270 non‐amnestic), with confirmed Aβ status (247 Aβ+; 346 Aβ‐; cerebrospinal fluid/positron emission tomography/autopsy) and single molecule array plasma measurements. Logistic regression and receiver operating characteristic (ROC) area under the curve (AUC) tested discrimination of Aβ+ from Aβ‐; K‐folds cross‐validation and exhaustive selection determined optimal biomarker combinations. Chi‐square tests compared correct classifications to errors of the multivariable model and plasma p‐tau_181_ across phenotype. Survival analyses tested time to global clinical dementia rating (CDR) progression.

**Results:**

Multivariable models (p‐tau+GFAP+NfL) had the best performance to detect Aβ+ in NormCog (ROCAUC=0.87) and were significantly better in Impaired (ROCAUC=0.87) compared to single analytes (all p<0.018) (Table 1; Figure 1). In ImpCog, multivariable model classification differed by phenotype (χ^2^=7, p=0.032), with highest accuracy in amnestic dementia (Accuracy: 92%), followed by MCI/impaired not MCI (81%), and non‐amnestic dementia (76%). Likewise, plasma p‐tau_181_ performance significantly differed by phenotype (χ^2^=6.8, p=0.035) (Accuracy: amnestic=90%; MCI/impairment without MCI=80%; non‐amnestic=74%). Survival analyses demonstrated that higher NfL best predicted faster CDR progression for both Aβ+ (HR=2.94, p=8.1e‐06) and Aβ‐ individuals (HR=3.11, p=2.6e‐09) (Figure 2).

**Conclusion:**

Combining plasma biomarkers can optimize detection of AD pathology across cognitively normal and clinically diverse neurodegenerative disease. Still, diagnostic accuracy may be lower for non‐amnestic AD. Plasma NfL showed the most accurate prognosis across neurodegenerative spectrum.